# Activity-Dependent Regulation of Alternative Cleavage and Polyadenylation During Hippocampal Long-Term Potentiation

**DOI:** 10.1038/s41598-017-17407-w

**Published:** 2017-12-12

**Authors:** Mariana M. Fontes, Aysegul Guvenek, Riki Kawaguchi, Dinghai Zheng, Alden Huang, Victoria M. Ho, Patrick B. Chen, Xiaochuan Liu, Thomas J. O’Dell, Giovanni Coppola, Bin Tian, Kelsey C. Martin

**Affiliations:** 1 Department of Biological Chemistry, David Geffen School of Medicine, University of California, Los Angeles, Los Angeles, CA USA; 2 Graduate Program in Areas of Basic and Applied Biology, University of Porto, Porto, Portugal; 3 Department of Microbiology, Biochemistry and Molecular Genetics, Rutgers New Jersey Medical School, Newark, NJ USA; 4 Department of Psychiatry and Biobehavioral Sciences, Semel Institute for Neuroscience, David Geffen School of Medicine, University of California, Los Angeles, Los Angeles, CA USA; 5 Interdepartmental Graduate Program in Neuroscience, University of California, Los Angeles, Los Angeles, CA USA; 6 Department of Physiology, David Geffen School of Medicine, University of California, Los Angeles, Los Angeles, CA USA

## Abstract

Long-lasting forms of synaptic plasticity that underlie learning and memory require new transcription and translation for their persistence. The remarkable polarity and compartmentalization of neurons raises questions about the spatial and temporal regulation of gene expression within neurons. Alternative cleavage and polyadenylation (APA) generates mRNA isoforms with different 3′ untranslated regions (3′UTRs) and/or coding sequences. Changes in the 3′UTR composition of mRNAs can alter gene expression by regulating transcript localization, stability and/or translation, while changes in the coding sequences lead to mRNAs encoding distinct proteins. Using specialized 3′ end deep sequencing methods, we undertook a comprehensive analysis of APA following induction of long-term potentiation (LTP) of mouse hippocampal CA3-CA1 synapses. We identified extensive LTP-induced APA changes, including a general trend of 3′UTR shortening and activation of intronic APA isoforms. Comparison with transcriptome profiling indicated that most APA regulatory events were uncoupled from changes in transcript abundance. We further show that specific APA regulatory events can impact expression of two molecules with known functions during LTP, including 3′UTR APA of *Notch1* and intronic APA of *Creb1*. Together, our results reveal that activity-dependent APA provides an important layer of gene regulation during learning and memory.

## Introduction

Long-term potentiation (LTP) is a form of synaptic plasticity that corresponds to a long-lasting increase in synaptic transmission in response to specific patterns of neuronal firing or activity, and underlies learning and memory^1,2^. The early-phase of LTP (E-LTP) is independent of new gene expression while the late-phase of LTP (L-LTP), which lasts several hours to days, requires new transcription and translation^3–6^.

Pre-mRNA cleavage and polyadenylation (C/P) is a nearly universal 3′ end processing mechanism for protein-coding genes in eukaryotes, and is coupled to transcription termination^7^. C/P consists of an endonucleolytic cleavage of pre-mRNAs followed by the synthesis of a polyadenosine tail, and is carried out by the C/P complex, which contains over 20 factors and many associated factors^8^. The site for C/P, known as polyA site (PAS), is defined by upstream and downstream cis regulatory elements, the most prominent of which is the A[A/U]UAAA element located upstream of the PAS^9–11^.

Most mammalian genes contain multiple PASs that yield multiple mRNA isoforms^12–14^. While the majority of alternative PASs are located within the 3′-most exon and lead to changes in 3′UTR lengths, a sizable fraction of PASs are located in introns and control the selection of alternative terminal exons, affecting both coding sequences (CDSs) and 3′UTRs^12^. A growing number of mechanisms have been found to regulate APA, including core C/P factors^15,16^, splicing factors^15,17^, and RNA-binding proteins that interact with sequence motifs near the PAS^18^.

Several tissues exhibit unique patterns of APA regulation^19–21^. For example, distal PASs tend to be selected in the brain, leading to preferential expression of mRNAs with long 3′UTRs^22–26^. Since the 3′UTR contains binding motifs for RNA-binding proteins and miRNA target sites, alteration of 3′UTR length offers an effective means to modulate gene expression by controlling aspects of mRNA metabolism, such as stability and translation^10,27–29^.

In keeping with the preferential expression of distal PAS isoforms in neurons, 3′UTRs play a particularly important role in compartmentalized gene expression by directing the localization and regulated translation of mRNAs within dendrites and axons and at synapses^30–33^. RNA sequencing of the synaptic neuropil in mouse hippocampus identified over 2,000 axonally and dendritically localized mRNAs^34^. Localization of mRNAs in neurons often depends on specific cis-acting elements within the 3′UTR^35,36^. Interestingly, Taliaferro *et al*. found that transcripts using distal alternative last exons tended to be localized to neurites^37^. In addition, several studies have reported APA regulation following neuronal activation. An early microarray analysis found that a set of genes expressed truncated mRNAs through APA in cultured rat neuronal hippocampal cells following chronic potassium chloride depolarization^38^. It was suggested that the APA events may couple with transcriptional regulation through MEF2^38^. Using Rat PC12 and mouse MN-1 neurons, Berg *et al*. showed that activity-induced APA changes were recapitulated by functional inhibition of U1 snRNP^17^, a complex that is involved not only in 5′ splice site recognition but also in the inhibition of premature usage of PAS^15,17,39^. The authors suggested that shortage of U1 snRNP during transcriptional upregulation following neuronal activation may lead to activation of proximal PAS.

Here, using deep sequencing of 3′ ends of transcripts, we systematically characterized APA regulation following LTP induction in acute mouse hippocampal slices. We examined both 3′UTR APA and intronic APA events at different time points post LTP, and analyzed the interplay between APA and gene expression regulation. Our study reveals global shortening of 3′UTR and activation of intronic APA, in line with previous reports. However, APA events are largely uncoupled from gene expression changes, and constitute a distinct layer of gene regulation during LTP.

## Results

**Figure 1:**
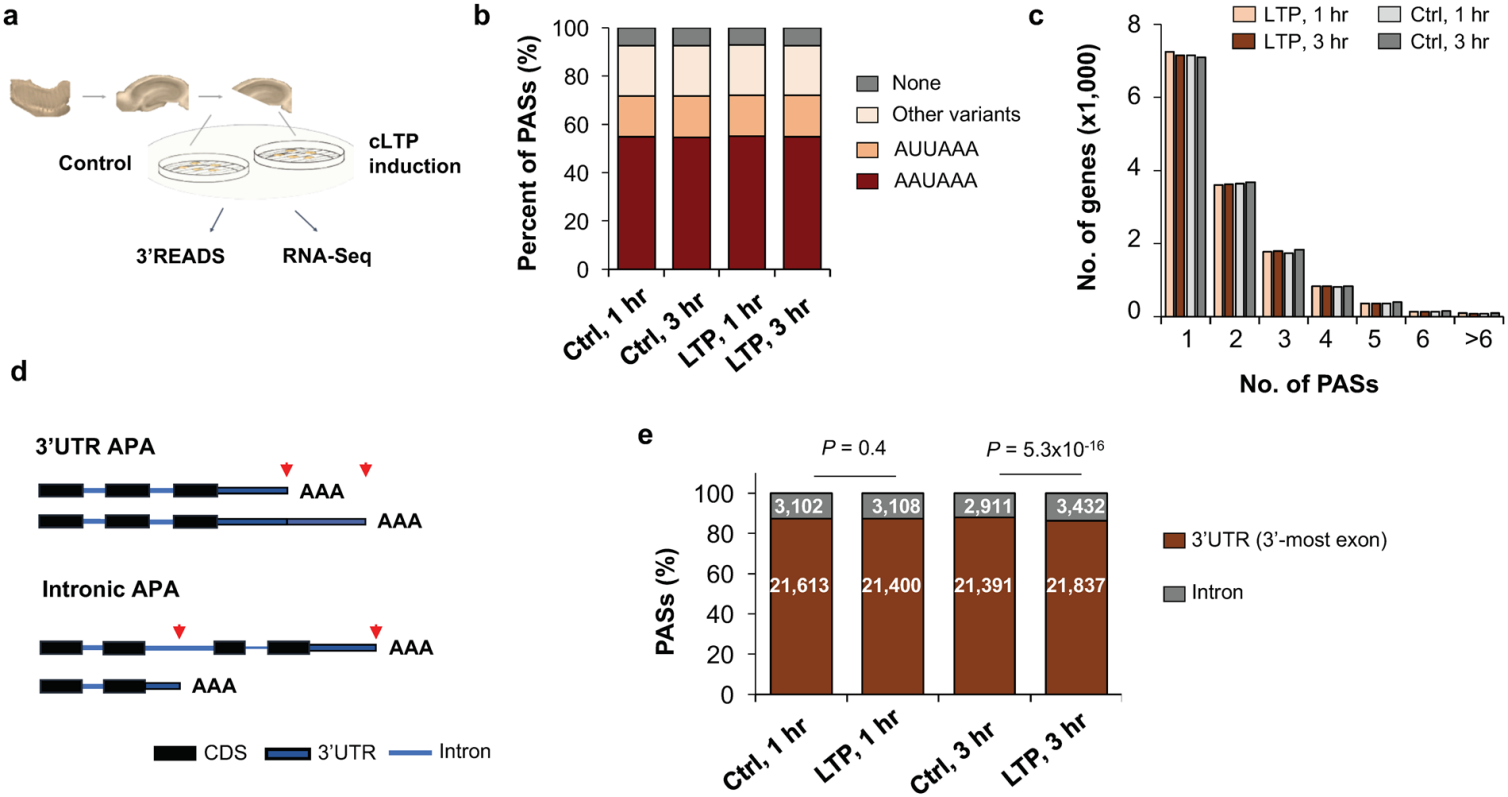
Analysis of APA in LTP. (**a**) Experimental design. RNAs from chemical LTP (cLTP)-induced and time-matched control hippocampal slices were subjected to 3′READS+ analysis to examine APA, or RNA-seq for gene expression. (**b**) Distribution of the A[A/U]UAA element in identified poly(A) sites (PASs). Percentage of PASs associated with either AAUAAA, AUUAAA, other variants of A[A/U]UAAA, or not associated with any A[A/U]UAAA element are shown for each sample group. (**c**) Number of PASs identified per gene. Genes with only one PAS have the highest frequency and the majority of genes displayed APA in the hippocampal samples analyzed. (**d**) Diagram showing 3′UTR APA (top) and intronic APA (bottom). 3′UTR APA isoforms have alternative 3′ UTRs resulting from the choice of different PASs in the 3′UTR. Two PASs are shown, i.e., proximal PAS and distal PAS. Intronic APA isoforms have different 3′UTRs as well as coding sequences (CDS). (**e**) Distribution of PASs in different regions of the mRNA: 3′UTR (in 3′-most exon only) vs. upstream intron. Change of PAS distribution during LTP is observed 3 hr post LTP induction. Number of PAS in each region is specified in each bar. P-values (Binomial test) indicate difference in fractions of 3′UTR PASs and intronic PASs in LTP vs. control samples.

We were interested in understanding whether and how APA, a widespread pre-mRNA processing mechanism, plays a role in LTP. To this end, we perfused acute hippocampal mini-slices with forskolin and high concentrations of calcium and potassium to induce chemical LTP (cLTP). This form of LTP depends on bursting of CA3 neurons and produces a long-lasting, NMDAR-dependent plasticity that requires new transcription and translation^40,41^. We extracted RNA from hippocampal mini-slices 1 (1 hr) and 3 hours (3 hr) post LTP induction (Fig. 1a), collecting time-matched controls from the same animals (Fig. 1a, see Materials and Methods^40,41^. Reverse transcription-quantitative PCR (RT-qPCR) analysis of the immediate early gene *Arc* as well as the short and long intronic APA *H* *omer1* isoforms confirmed their regulation after LTP induction, as previously reported^42,43^.

To determine APA profiles after LTP induction, we subjected RNA samples to 3′READS, a method we previously developed to specifically study the 3′ end of transcripts^44^ (see Materials and Methods). After exclusion of outlier samples (Fig. S1), we obtained >10 million (M) PAS-containing reads per sample and identified 24,908 PASs in 13,445 genes, including 294 non-coding RNA genes. About 55% of identified PASs were associated with AAUAAA, 17% with AUUAAA, 20% with other close variants, and ~8% with no identifiable A[A/U]UAAA or their close variants in the −40 to −1 nt region of the PAS (Fig. 1b). These values were comparable at 1 hr and 3 hr, for both control and LTP samples. We note that a higher percentage of PASs were associated with the AAUAAA hexamer in our RNA samples than the 42% previously reported in the mouse genome^12^, presumably because APA transcripts in brain preferentially use distal PASs^4,22,24–26^, which are more frequently associated with AAUAAA than proximal PASs^13^.

Approximately half of all detected protein-coding genes used 2 or more PASs, with no significant difference in the number of PASs per gene between control and LTP samples (Fig. 1c). As shown in Fig. 1e, most PASs (>85%) were found in the 3′UTR of the 3′-most exon, with less than 15% of PASs located in introns. Notably, we observed a small but significant (3,432 vs. 2,911, *P* = 5.3 × 10^−16^, Binomial test) global increase in the number of detected intronic PASs 3 hr post LTP, suggesting upregulation of intronic PAS usage following LTP induction (see below for more analysis). By contrast, no significant difference was detected 1 hr post LTP.

### LTP induces global shortening of 3′UTRs

**Figure 2:**
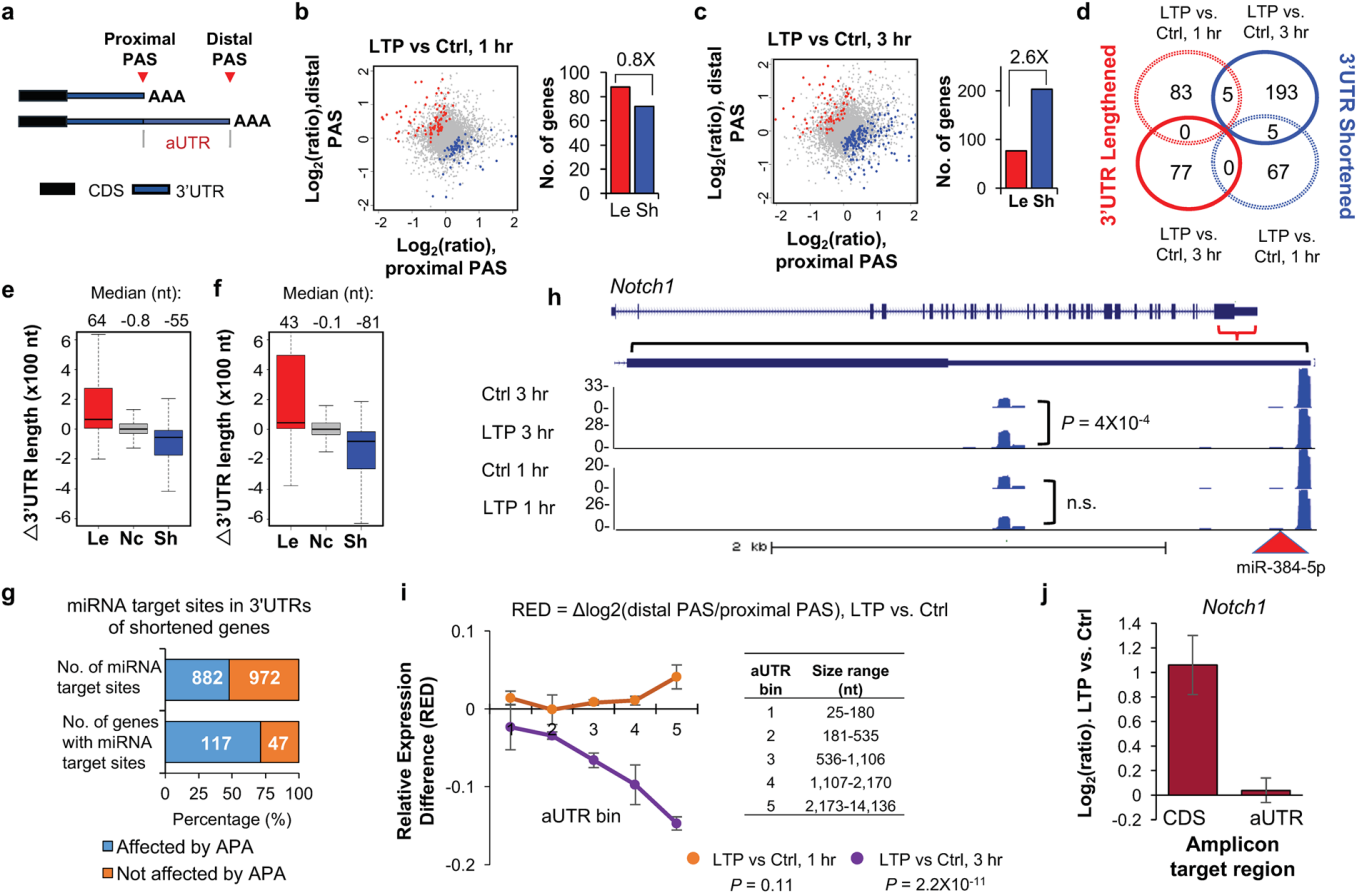
Regulation of 3′UTR APA after LTP induction. (**a**) Schematic of 3′UTR APA. (**b**) 3′UTR APA regulation after 1 hr LTP induction. Left, Scatterplot comparing expression changes of proximal and distal PASs after 1 hr LTP induction. Genes that significantly switched to proximal PAS usage are in blue and those that switched to distal PAS usage are in red (P < 0.05, DEXSeq, and relative abundance change >5%). Grey dots are genes without significant APA regulation. Right, bar graph comparing the number of genes with lengthened or shortened 3′UTRs (Le and Sh, respectively). (**c**) As in (**b**), 3 hr post LTP induction. (**d**) Venn diagram comparing genes with significant 3′UTR regulation 1 hr post LTP induction and 3 hr post LTP induction. (**e**) 3′UTR length change in 1 hr post LTP induction. Genes with 3′UTRs significantly shortened (blue), lengthened (red), or unchanged (grey) are shown. 3′UTR size was based on weighted mean of all 3′UTR isoforms. Median values are indicated on the top. (**f**) As in (**e**), except that data are based on 3 hr post LTP induction. (**g**) Effect of 3′UTR shortening on miRNA targeting. Number of miRNA target sites that are removed (882) or not removed (972) by 3′UTR shortening is indicated in the upper bar, and number of genes that contain removed (117) or not removed (47) miRNA target sites by 3′UTR shortening are indicated in lower bar. (**h**) An example gene *Notch1*, which displayed 3′UTR shortening after LTP. UCSC genome browser tracks of 3′READS data are shown. Gene structure and 3′UTR sequence are indicated on top. Peaks from two polyA sites indicate reads for corresponding APA isoforms. miR-384-5p target site is indicated. Reads are based on combined samples. (**i**) Relationship between aUTR size and 3′UTR-APA regulation at 1 hr or 3 hr post LTP induction. Relative expression difference (RED) was calculated for all genes with APA sites. The formula of RED is indicated above the graph. Genes were divided into five groups (aUTR bins, listed on the right) based on aUTR size, with approximately equal number of genes in each bin. For each bin, mean RED was calculated. Error bars are standard error of mean (SEM). P-value (Wilcoxon rank sum test) indicates the difference between bin 1 and bin 5. (**j**) RT-qPCR validation of *Notch1* APA regulation. Two different primer sets were used to detect the expression of a common coding region (left bar) and a region in the alternative 3′UTR (right bar). Error bars are SEM of 4 replicates.

We next focused on 3′UTR APA events and asked whether there was a global change in 3′UTR length after LTP induction. To simplify our analysis, we focused on the two most abundant 3′UTR APA isoforms, named proximal and distal PASs based on their relative positions in the 3′UTR (Fig. 2a), and compared their relative expression changes in LTP-induced vs. control samples. While similar numbers of genes displayed 3′UTR shortening and lengthening after 1 hr of LTP induction (72 vs. 88, Fig. 2b), a bias toward 3′UTR shortening was detected 3 hr post LTP induction (203 vs. 77, Fig. 2c). Notably, the genes that displayed 3′UTR changes 3 hr after LTP induction were largely distinct from those with 3′UTR changes 1 hr after LTP induction (Fig. 2d).

To measure the extent of 3′UTR length change elicited by LTP, we divided genes into groups with lengthened 3′UTRs, unchanged 3′UTRs or shortened 3′UTRs, and calculated their average difference in 3′UTR length. At 1 hr, both lengthened and shortened 3′UTR genes underwent mild changes in 3′UTR length (median = 64 nucleotides (nt) and 55 nt, respectively, Fig. 2e). By contrast, at 3 hr, while genes with lengthened 3′UTRs displayed a mild change of 3′UTR length (median = 43 nt), genes with shortened 3′UTRs showed a more pronounced 3′UTR length change (median = 81 nt) (Fig. 2f).

3′UTR length changes have previously been shown to alter miRNA targeting^45,46^. We next set out to globally identify miRNA target sites that would be affected by 3′ UTR shortening 3 hr after LTP induction. Of the 164 genes that displayed both 3′UTR shortening and contained miRNA target sites (based on the TargetScan database), 117 had their miRNA target sites (882 sites in total) removed by 3′UTR shortening (Fig. 2g). Thus, 3′UTR shortening could potentially play an important role in modulating miRNA regulation following LTP induction. An example gene, *Notch1*, is shown in Fig. 2h, displayed significant 3′UTR shortening in the 3 hr samples (*P* = 4 × 10^−4^, Fig. 2e), but not in the 1 hr samples. The Notch1 protein has been reported to be critical for hippocampal synaptic plasticity and memory formation^47,48^. Interestingly, we found a target site for miR-384-5p between the proximal and distal PASs of *Notch1* (Fig. 2h), a miRNA whose downregulation was shown to be required for the maintenance of LTP^49^. Thus, shortening of *Notch1* 3′UTR after LTP induction could help de-repress *Notch1* expression by miR-384-5p, contributing to LTP maintenance. RT-qPCR analysis using primer sets targeting a common coding region and a region that exists only in the long 3′UTR isoform confirmed increased *Notch1* gene expression and relatively higher expression of short 3′UTR isoform compared to long 3′UTR isoform (Fig. 2j).

Previous studies in other systems have indicated that the distance between two 3′UTR PASs, also known as alternative 3′UTR (aUTR) size (Fig. 2a), often correlates with the extent of 3′UTR APA^15^, a phenomenon likely attributable to competition between the two adjacent PASs for usage. We thus divided genes with 3′UTR-APA regulation at 1 hr or 3 hr post-LTP into five groups based on their aUTR sizes (aUTR bins 1–5) and asked whether the difference in the relative expression of two APA isoforms between LTP and control samples (relative expression difference, RED) was a function of aUTR size. The mean RED values shown in Fig. 2i revealed that genes with longer aUTRs underwent significantly greater 3′UTR shortening 3 hr post-LTP as compared to 1 hr (gene bin 1 vs gene bin 5 comparison at 3 hr: p-value = 2.2 × 10^−11^, Wilcoxon rank sum test). Together, these results indicate that LTP drives a general shortening of 3′UTR 3 hr post LTP induction, especially in transcripts with long aUTRs.

### 3′UTR regulation and changes in transcript abundance

**Figure 3:**
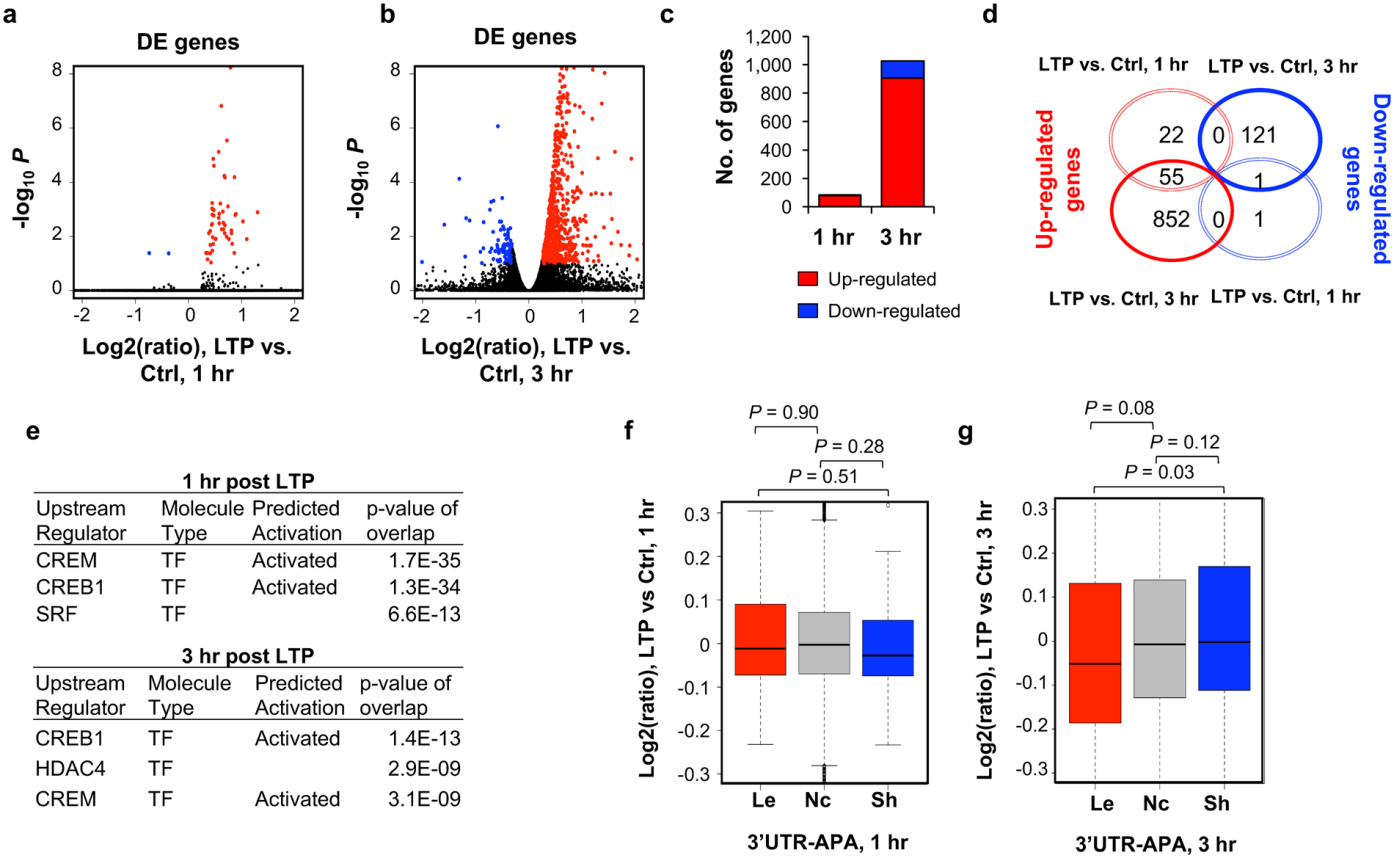
Regulation of gene expression after LTP induction. Differentially expressed (DE) genes 1 hr (**a**) or 3 hr (**b**) post LTP induction, as determined by RNA-seq. X-axis, log2(ratio) of gene expression (LTP vs Control); Y- axis, −log_10_ *P* (DESeq). DE genes were selected based on expression change >20% and FDR <0.1. Genes with upregulated expression are highlighted in red and those with downregulated expression in blue. Black dots represent genes without significant regulation. (**c**) Bar graph summarizing DE genes shown in (**a**) and (**b**). (**d**) Venn diagram comparing DE genes at 1 hr LTP and 3 hr LTP. (**e**) IPA upstream regulator analysis for significantly regulated genes. This data is generated through the use of Ingenuity Pathways Analysis, a web-delivered application (www.Ingenuity.com). (**f**) and (**g**) Gene expression regulation vs. LTP-induced 3′UTR-APA regulation. Box plots showing the log2(ratio) of gene expression, LTP vs. Ctrl, for genes with 3′UTR lengthened (Le), shortened (Sh) or no change (Nc) at 1 hr (**f**) or 3 hr (**g**) post LTP induction. Only genes with significant 3′UTR APA regulation (those from Fig. 2) were included. P-values (Wilcoxon rank sum test) indicate difference between gene groups.

Gene ontology analysis of genes with 3′UTR shortening indicated that genes with diverse functions are affected by this mechanism (Table S3). We next asked whether there was any correlation between LTP-induced 3′UTR changes and LTP-induced changes in transcript abundance. To obtain high resolution gene expression data, we performed RNA-seq from a separate set of acute mouse hippocampal mini-slices 1 hr or 3 hr after LTP induction, with time-matched controls from the same animals. To prevent 3′UTR changes from influencing gene expression analysis, we used only RNA-seq reads mapping to the coding region of genes (see Materials and Methods for details). We identified 79 and 1,029 genes that were significantly differentially expressed at 1 hr and 3 hr time points (fold change >1.2, FDR <0.1, DEseq) (Fig. 3a and b). At both time points, upregulated genes outnumbered downregulated ones (77 vs. 2 at 1 hr; 907 vs. 122 at 3 hr, Fig. 3c), indicating that LTP primarily elicits activation of gene expression^42^. Notably, most genes regulated at 1 hr were also regulated at 3 hr (Fig. 3d), indicating continuous activation of expression. The top differentially expressed (DE) genes we identified are consistent with previous studies^50–52^, including upregulation of *Gadd45g*, *Egr2*, *c-Fos*, *Arc* and *Npas4* (Table 1). Notably, gene expression changes based on RNA-seq were well correlated with those based on 3′READS (r = 0.81 and r = 0.78 for significantly regulated genes at 1 hr and 3 hr, respectively, Figure S2), attesting to the quality of our sequencing data.

**Table 1:** Top 25 differentially expressed (DE) genes at 1 hr and 3 hr post LTP induction.

**DE genes 1 hr post LTP**			**DE genes 3 hr post LTP**		
**Gene**	**Log2(ratio)**	**FDR**	**Gene**	**Log2(ratio)**	**FDR**
*Btg2*	2.4	2.5E-99	*Rasl11a*	2	2.7E-86
*Egr2*	2.3	2.1E-80	*Fos*	1.5	2.4E-64
*Fos*	1.9	3.5E-70	*Tinf2*	1.6	1.1E-51
*Nr4a1*	1.6	8.8E-66	*Arc*	1.2	2.1E-42
*Arc*	1.8	1.3E-47	*Cyr61*	1.8	1.4E-41
*Dusp6*	1.0	3.9E-27	*Txndc11*	1.1	2.9E-32
*Gadd45g*	1.3	1.4E-26	*Sik1*	1.3	7.7E-32
*Npas4*	2.6	2.7E-22	*Thbs1*	1.8	2.3E-31
*Dusp1*	1.3	3.3E-19	*Gem*	1.1	4.9E-26
*Trib1*	1.3	2.2E-18	*Btg2*	1.2	3.7E-25
*Ppp1r15a*	1.0	1.0E-17	*Pax6*	1.1	1.3E-22
*Errfi1*	0.8	2.6E-17	*Gadd45g*	1.0	1.3E-21
*Egr1*	1.3	7.7E-16	*Il6*	1.2	8.1E-20
*Nr4a2*	0.9	1.0E-15	*Kmt2d*	0.8	2.0E-19
*Arl4d*	1.1	4.3E-15	*Trh*	2.1	3.4E-19
*Sik1*	1.0	3.9E-14	*Rtl1*	0.8	1.6E-17
*Fosb*	1.4	1.0E-13	*Ppp1r3g*	1.4	7.7E-16
*Rgs4*	0.7	1.8E-13	*Grin2b*	0.7	1.3E-15
*Egr4*	2.1	7.8E-13	*Rnf217*	0.9	6.2E-14
*Ciart*	0.9	3.4E-12	*Ccnl1*	0.7	7.1E-13
*Cyr61*	0.8	4.3E-10	*Nfkb1*	0.7	1.1E-12
*Csrnp1*	0.7	4.3E-10	*Nfil3*	0.8	1.1E-12
*Thbs1*	0.9	7.7E-10	*Sipa1l3*	0.9	2.2E-12
*Junb*	1.7	9.0E-10	*Nr4a2*	1.0	8.0E-12
*Ptgs2*	0.9	1.7E-09	*Iqsec2*	0.7	8.0E-12

Log_2_(ratio) and FDR (DESeq). FDR was based on multiple testing adjustment using the Benjamini-Hochberg method.

GO analysis of DE genes after LTP induction revealed several shared terms at 1 hr and 3 hr, including those related to signaling (“signal transduction” and “single organism signaling”), metabolic process (“positive regulation of metabolic process” and “negative regulation of macromolecule metabolic process”, “nucleic acid metabolic process”), and nucleus (“nucleus”, Table 2). Additionally, DE genes at 1 hr were enriched in “tissue development” and “transcription factor complex” (Table 2). At 3 hr, DE genes were also enriched in “neuron projection development” and “excitatory synapse” (Table 2). Thus, GO terms do not appear to overlap with those associated with genes showing 3′UTR shortening (Table S3). In addition, Ingenuity Pathway Analysis (IPA) identified a set of transcription factors (TFs) that are predicted to regulate genes with expression changes post LTP (Fig. 3e), including CREM, CREB1, and HDAC4, which is consistent with the notion that CREB proteins play key roles in memory and synaptic plasticity, and facilitate the late phase of LTP^53–55^. By contrast, no significant TFs were predicted to be associated with genes showing 3′UTR shortening (data not shown). Taken together, both GO and IPA analysis results indicate that transcriptional regulation and APA may target different sets of genes.

**Table 2:** GO terms enriched for upregulated and downregulated genes 1 hr or 3 hr post LTP induction.

**GO term**	**Category**	**−Log**_**10**_ ***P***
**Enriched for DE genes, 1 hr post LTP**		
positive regulation of metabolic process	BP	16.3
negative regulation of macromolecule metabolic process	BP	12.1
tissue development	BP	10.6
signal transduction	BP	8.8
nucleic acid metabolic process	BP	8.6
nucleus	CC	8.4
transcription factor complex	CC	4.0
protein phosphatase type 1 complex	CC	3.0
**Enriched for DE genes, 3 hr post LTP**		
neuron projection development	BP	7.5
single organism signaling	BP	7.3
regulation of nucleic acid-templated transcription	BP	5.9
negative regulation of macromolecule metabolic process	BP	5.5
positive regulation of metabolic process	BP	5.5
neuron part	CC	8.6
excitatory synapse	CC	4.6
nucleus	CC	3.9

BP, biological process; CC, cellular component. *P* is based on the Fisher’s exact test.

To specifically address the interplay between 3′UTR-APA change and gene expression regulation, we analyzed the mRNA expression fold change of genes with significantly shortened and lengthened 3′UTR isoforms (P < 0.05, DEXSeq and relative abundance change of APA isoform >5%). There was no discernable correlation between gene expression change and 3′UTR-APA regulation at 1 hr post LTP (Fig. 3f). At 3 hr post LTP, while genes with lengthened 3′UTRs appeared to be modestly downregulated as compared to genes with shortened or unchanged 3′UTRs (P = 0.03 or P = 0.08, respectively, Wilcoxon test, Fig. 3g), there was no discernable difference between genes without 3′UTR APA changes and those with shortened 3′UTRs (P = 0.12, Wilcoxon test, Fig. 3g). Therefore, alteration of 3′UTR length is largely uncoupled from gene expression changes.

### Widespread activation of intronic APA during LTP

**Figure 4:**
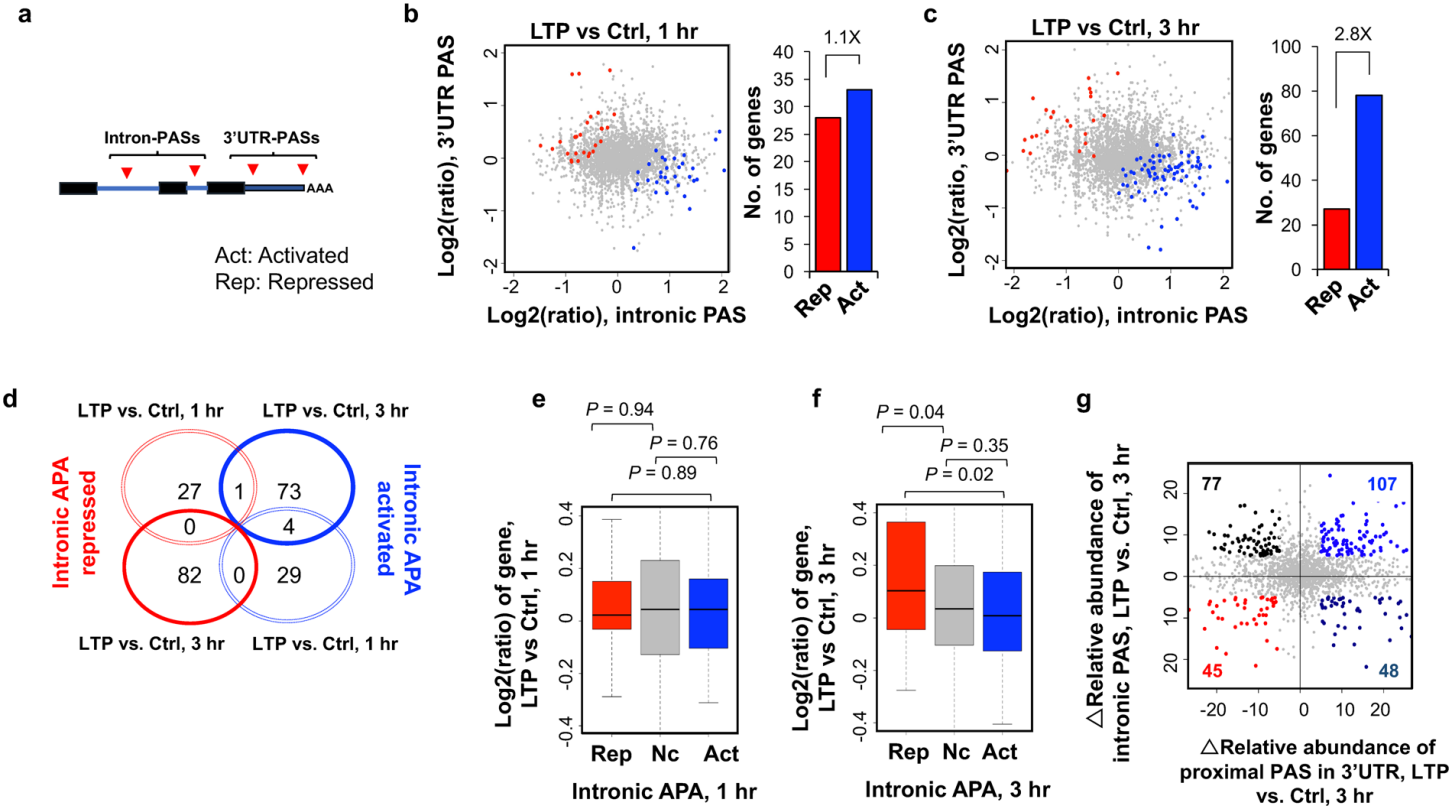
Regulation of intronic APA after LTP induction. (**a**) Schematic of intronic APA analysis. Intronic PASs were compared with 3′UTR-PASs. (**b**) and (**c**) Left, scatterplot comparing expression change (LTP vs. control) of intronic PASs (x-axis) and 3′UTR PASs (y-axis). Genes with significantly activated intronic PAS usage are shown in blue (Act) and those with significantly repressed intronic PAS usage in red (Rep) (p < 0.05, DEXSeq and relative abundance change >5%). Grey dots represent genes without significant regulation (Nc). Right, bar graph showing the number of genes with activated or repressed intronic APA. (**d**) Venn diagram comparing genes with CDS-APA regulation at 1 hr post LTP and 3 hr post LTP. Genes correspond to those in (**b**) and (**c**). (**e**) and (**f**), Box plot showing the log_2_ratio (LTP/Ctrl) of genes with intronic PAS activation or intronic PAS repression, 1 hr (**e**) or 3 hr (**f**) post LTP induction. Genes with significant intronic APA regulation are those from (**b**) or (**c**). P-values (Wilcoxon rank sum test) indicate the difference between groups. (**g**) Scatterplot comparing 3′UTR APA regulation (x-axis) and intronic APA regulation (y-axis) at 3 hr post LTP induction. Δ relative abundance for 3′UTR APA is based on the two 3′UTR PASs with most reads, as in Fig. 2, and Δ relative abundance for intronic APA is based on all intronic PAS reads vs. all 3′UTR PAS reads. Genes with intronic APA and/or 3′UTR APA change (>5% relative abundance) are highlighted with different colors based on the type of change.

About 12% of APA sites identified in our samples were located in introns (Fig. 1e), which can impact coding sequence usage. We next tested the significance of the LTP-induced regulation of intronic APA usage. We combined all isoforms using PASs in RefSeq-supported introns and compared their expression with all transcripts using PASs in the 3′-most exon (Fig. 4a). We detected a modest difference at 1 hr between genes with activated intronic APA and genes with repressed intronic APA (33 vs. 28, Fig. 4b). However, at 3 hr post LTP, genes with activated intronic APA significantly outnumbered those with repressed intronic PAS by 2.8-fold (78 vs. 27, Fig. 4c). These data indicate that LTP induces not only a general shortening of 3′UTR but also triggers an overall transcript truncation through increased usage of intronic PASs. In addition, the genes with intronic APA regulation at 1 hr differed from those with intronic APA regulation at 3 hr (Fig. 4d).

We then asked whether intronic APA is related to gene expression level changes. At the 1 hr post LTP time-point, gene expression changes appeared to be unrelated to intronic APA regulation (Fig. 4e). By contrast, at 3 hr post LTP, genes with repressed intronic APA tended to be upregulated relative to genes without APA regulation or with activated intronic APA (P = 0.04 or 0.02, respectively, Wilcoxon rank sum test, Fig. 4f). However, genes with activated intronic APA, which accounted for most of the APA events, did not show significant difference in expression compared to genes without intronic APA changes (P = 0.35, Wilcoxon rank sum test). Therefore, intronic activation of APA is not coupled with activation of gene expression. GO analysis did not reveal any terms highly significantly enriched for genes with activated intronic APA (Table S4), indicating that intronic APA regulation, unlike transcriptional control, is not specific for genes with certain functions.

We next examined how intronic APA was related to 3′UTR-APA. Using the 3 hr post-LTP data, we identified 107 genes with both shortened 3′UTRs and activated intronic APA, a number greater than genes with shortened 3′UTRs and repressed intronic APA (48), lengthened 3′UTRs and activated intronic APA (77) or lengthened 3′UTRs and repressed intronic APA (45) (Fig. 4g). This result indicates that, for a group of genes, 3′UTR shortening is coupled with activation of intronic APA. Presumably, for those genes, proximal PASs are generally preferred regardless of the location, in the 3′-most exon or in an intron.

**Figure 5:**
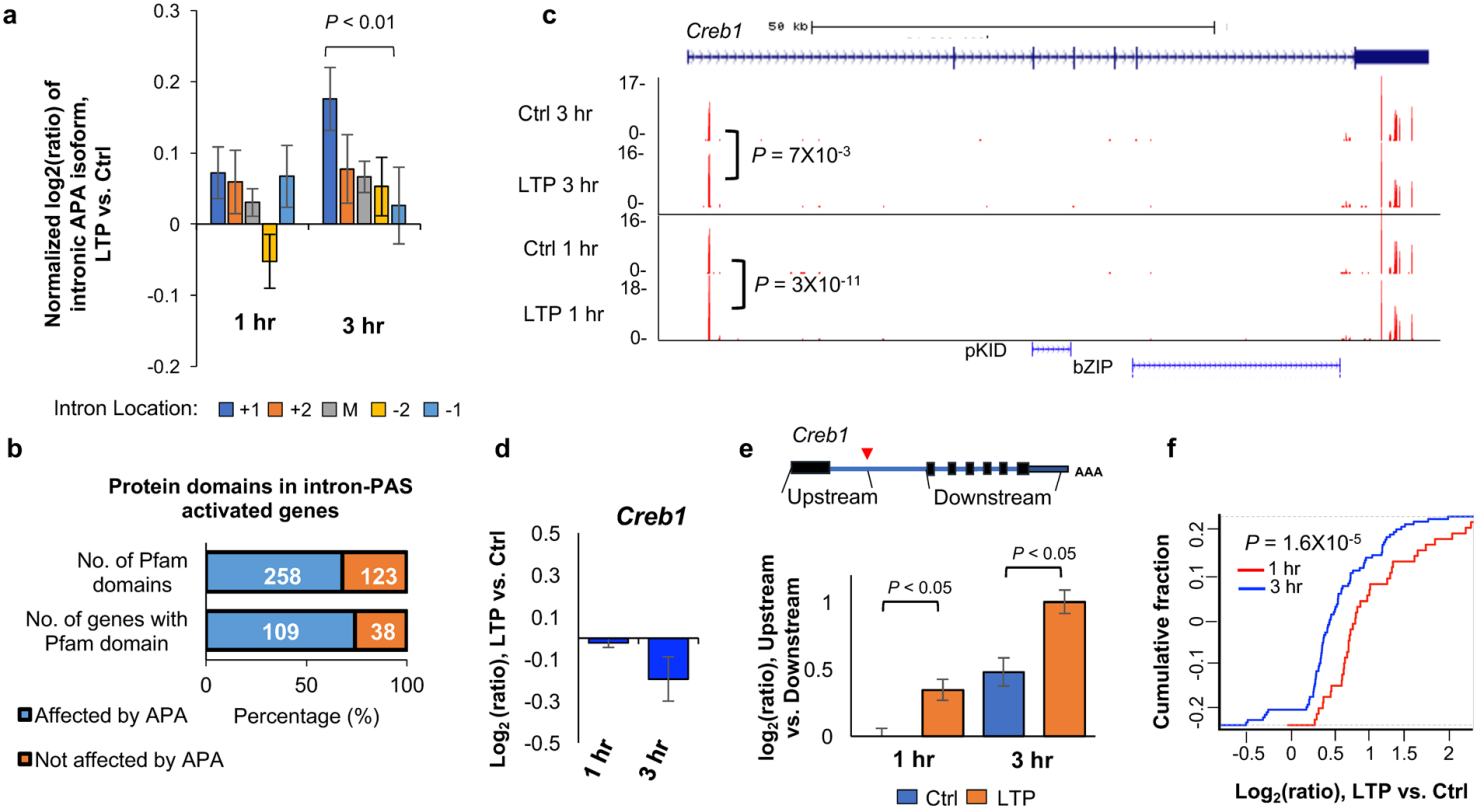
Characteristics of intronic APA after LTP induction. (**a**) Normalized expression changes of intronic PAS isoforms. Intronic PAS isoforms were divided into five groups based on the intron location where PAS resides, i.e., first (+1), second (+2), last (−1), second to last (−2), and middle (between +2 and −2 introns). Expression changes are expressed as log2(ratio), LTP vs. Ctrl. Only genes with ≥4 introns and only PAS isoforms with ≥2 reads were used for analysis. Values for five intron groups were normalized by mean-centering. Error bars are standard error of mean. P-value (Wilcoxon rank sum test) indicating difference between first and last intron values is shown. (**b**) Protein domains that can potentially be removed or truncated by intronic PAS activation. Number of pfam domains that can be removed or not removed by intronic APA is indicated in the upper bar, and number of genes that contain domains removed or not removed by intronic PAS activation is indicated in the lower bar. (**c**) An example gene *Creb1*, which displayed significant intronic PAS activation. Gene structure is shown on top and peaks for PASs are shown in UCSC genome browser tracks. P-values based on comparison of intronic PAS and 3′UTR PAS (DEXSeq) are indicated. Pfam domains are indicated. Reads are acquired by combining samples. (**d**) Gene expression change of *Creb1* after LTP. (**e**) Expression changes based on RNA-seq reads mapped to different regions of *Creb1*. Schematic of *Creb1* is shown on the top, and log2(ratio) from indicated region is shown in a bar plot. Two regions were analyzed, including the region from transcription start site to the intronic PAS, and the region from the second to the last exons. (**f**) Gene expression changes (LTP vs. Ctrl) of CREB1 target genes obtained from the IPA database. The blue curve corresponds to 81 target genes in the 3 hr post LTP samples, whereas the red corresponds to 37 target genes in the 1 hr post LTP samples.

Previous studies have shown that intronic APA events regulated by certain factors, such as U1 snRNP (U1)^17^ and RNA polymerase II associated factor (PAF) complex^56^, display a 5′ to 3′ polarity, i.e., more regulation at the 5′ end than the 3′ end. By dividing intronic PAS isoforms into 5 groups based on the intron locations of their PASs, i.e., first intron (+1), second (+2), last (−1), second to last (−2), and middle (between +2 and −2 introns), we found that PASs located in the 5′-most intron (+1) indeed had the highest increase in usage as compared to those in the 3′-most intron 3 hr post LTP (*P* = 0.008, Wilcoxon rank sum test). By contrast, no such trend was discernable with the 1 hr APA events (Fig. 5a). This result suggests that a regulatory mechanism similar to U1 and PAF complex, which specifically impacts 5′ intronic PASs, takes place 3 hr post LTP (see Discussion).

To further explore the consequences of intronic APA activation following LTP induction, we examined protein domains that could be removed or truncated through shortening of coding regions. Of the 147 genes that showed significant intronic PAS activation and contained Pfam-annotated domains, 109 could lose their protein domains (258 domains in total) as a result of intronic APA activation (Fig. 5b). One of the most significantly regulated genes by intronic APA was *Creb1*, whose intronic PAS increased both 1 hr and 3 hr post LTP inductions (Fig. 5c). The regulated intronic PAS is located at the 5′ end of the first intron (Fig. 5c), usage of which could remove much of the protein sequence of *Creb1*, including two key domains—the phosphorylated kinase-inducible-domain (pKID) and basic leucine zipper domain (bZIP) (Fig. 5c). Since *Creb1* plays a central role in transcriptional activation of many genes during LTP, as previously shown^57,58^ and predicted by our IPA analysis in this study (Fig. 3e), we hypothesized that activation of its intronic APA may function to inhibit *Creb1* expression, blunting its role in transcriptional activation. Indeed, using the RNA-seq data, we found that while *Creb1* mRNA appeared unchanged 1 hr post LTP, it was slightly downregulated 3 hr post LTP (Fig. 5d). Importantly, downregulation of *Creb1* mRNA mainly occurred to exons downstream of the intronic PAS but not to the first exon (Fig. 5e), suggesting that downregulation of *Creb1* mRNA level occurred through the usage of intronic PAS. Consistent with repressed *Creb1* expression, upregulation of *Creb1* target genes was decreased 3 hr post LTP as compared to 1 hr post LTP (*P* = 1.6 × 10^−5^, Fig. 5f). Notably, the intronic PAS of *Creb1* is conserved in human and rat based on PolyA_DB^59^, highlighting its functional importance. Taken together, intronic APA of Creb1 may play an important role in downregulation of its gene expression, controlling the extent and/or timing of the Creb1-mediated gene expression program.

## Discussion

Regulation of transcription and protein synthesis is critical during L-LTP^49,60–64^, and post-transcriptional gene regulation is key to the development and function of neural circuits^35,65,66^. mRNA isoform changes through APA represent a widespread, although poorly understood, mechanism in eukaryotes. APA may play a special regulatory role in the nervous system, because neuronal transcripts generally have long 3′UTRs^21,24^ and sequence motifs in 3′UTRs contribute to both spatial and temporal control of gene expression during neuronal plasticity^36^. Inspired by studies indicating activity-dependent regulation of APA in neurons^17,38^, we undertook a genome-wide approach to systematically examine activity-dependent APA regulation following LTP induction in mouse hippocampal slices. With a specialized 3′ end-based sequencing method, 3′READS, we uncovered a global trend of 3′UTR shortening and activation of intronic APA 3 hr post LTP, a time point that also involves substantial transcriptional changes. By contrast, the global APA regulation was not discernable 1hr post LTP, when only a small number of genes have expression changes. Thus, it is possible that regulation of PAS usage, a co-transcriptional process, is globally coupled with transcriptional regulation. On the other hand, for individual genes, we did not observe coupling between 3′UTR shortening or activation of intronic PASs with regulation of gene expression.

### Comparison with previous studies

The APA regulation following LTP induction detected in our study is consistent with a previous microarray study done in cultured rat hippocampal neurons that reported significant transcript truncation following KCl depolarization^38^. Of note, our stimulation protocol induced Hebbian synaptic plasticity^41^, while the 1 hr or 6 hr of continuous KCl depolarization in cultured neurons likely produced homeostatic plasticity^67^. Despite this difference, 10 of the 29 genes undergoing APA regulation (34.5%, 30 predicted-only genes from Flavell *et al*., 2008 were excluded) following KCl depolarization also underwent APA regulation after LTP induction in hippocampal slices, suggesting a common mechanism of APA regulation during homeostatic and Hebbian plasticity. Our high-resolution genome-wide approach provides additional insights into APA regulation during plasticity by expanding the catalog of genes with activity-dependent APA changes from a total of 59 in Flavell *et al*. (2008) to over 1,100 genes (Fig. 1f). Our study further provides a comprehensive characterization of the two different types of APA regulation, 3′UTR APA and intronic APA, which could not be dissected using microarrays or traditional RNA-seq. Notably, we also performed the same experiments using PAS-Seq, another 3′ end-based sequencing method^68^. However, due to high variance of read counts across samples and within genes, we could not identify significantly regulated APA isoforms following LTP induction (data not shown), attesting to the technical advantage of using 3′READS in analysis of APA.

### Potential mechanisms

The molecular mechanism(s) underlying activity-dependent APA regulation during LTP remain largely unclear. Global shortening of 3′UTRs and activation of intronic APA sites have been observed in proliferating cells compared to quiescent ones^45^. Cancer cells, undifferentiated cells and cells at early developmental stages display similar patterns^46,68,69^. The mechanisms underlying proliferation-based APA are largely unknown, although higher expression levels of C/P factors and thus increased general C/P activity have been suggested to be responsible for more efficient 3′ end processing at proximal PASs^69,70^. While we did not observe increased expression of C/P factors at the RNA level post LTP (Fig. S3a), the C/P factor gene *Wdr33* did appear to be regulated by APA (Fig. S3b), raising the possibility that regulation of certain C/P factors may lead to the global APA changes observed in this study. In addition, we cannot rule out the possibility that some of the C/P factors might be regulated post-translationally and have altered activities post LTP.

The Dreyfuss lab reported that a shortage of U1 snRNP (U1) relative to the pre-mRNA abundance caused subdued inhibition of cleavage/polyadenylation by U1^17^ and, consequently, activation of proximal PASs in introns and 3′UTRs. Indeed, using our previous 3′READS data from mouse C2C12 myoblast cells^15^, we found that the expression of intronic APA isoform of *Creb1* increased by 3.7-fold when U1 was functionally inhibited by an antisense oligo to U1 snRNA (Fig. S5a). Global activation of proximal PASs in 3′UTRs and introns has also been reported in cells with reduced expression of the PAF complex^56^, which plays a role in promoter-proximal pausing and transcriptional elongation^71,72^. We also re-analyzed our previous data of knockdown of Paf1, one of the subunits of PAF complex, in C2C12 cells. Interestingly, intronic APA of *Creb1* increased by 7.8-fold when Paf1 was knocked down (Fig. S5b). Although we did not detect a global correlation in intronic APA regulation between U1 inhibition or Paf1 knockdown and LTP activation, there were modest correlations between these conditions for PAS regulation in the first intron (r = 0.21 and 0.31 for Paf1 knockdown vs. LTP and for U1 inhibition vs. LTP, respectively, Pearson correlation, Fig. S5d and Fig. S5e). This result suggests that PASs in the first intron, as in the case of *Creb1*, might be regulated by U1 and/or PAF mechanisms in cells activated for LTP. Future studies need to determine mechanistic details concerning these potential connections.

Intriguingly, we also found from our previous data that knockdown of PABPN1 expression by siRNA (siPABPN1) in C2C12 cells substantially upregulated the expression of the intronic APA isoform of *Creb1* by 16-fold in C2C12 cells^15^. Because of PABPN1′s role in nuclear RNA surveillance^15,73^, it is possible that the intronic PAS isoform of *Creb1* is rapidly degraded after usage. This further supports the model that intronic polyadenylation of *Creb1* serves to inhibit the expression of full-length isoform. Moreover, similar to U1 inhibition and Paf1 knockdown, regulation of PASs in the first introns of genes by LTP was modestly correlated with those by siPABPN1 (r = 0.34, Pearson correlation, Fig. S5f), suggesting a general pattern similar to that of the intronic PAS of *Creb1*.

### Functional implications

APA generates mRNA isoforms with different 3′UTR lengths and/or coding sequences. mRNA isoforms resulting from LTP induction may localize to distinct subcellular compartments, and produce different protein levels^23,28,74^. The functional consequences of 3′UTR-APA events emerge from differences in cis-regulatory elements contained within the alternative 3′UTRs, including motifs recognized by miRNAs and RBPs. Shorter 3′UTR isoforms can escape miRNA-mediated destabilization and translation repression through the loss of miRNA binding sites, a strategy that is used to achieve both cell type specificity and correct developmental timing^75–77^. Indeed, we found that genes with lengthened 3′UTRs tended to be downregulated, implying a role for 3′UTR in gene regulation during hippocampal plasticity. A case in point is the *Notch1* gene, which plays important roles in LTP and whose 3′UTR shortening removes a target site of miR-384–5p, an important miRNA for LTP maintenance^47–49^. Future studies are needed to examine the detailed mechanisms and consequences behind APA of *Notch1* and explore other similar cases.

Intronic APA events can impact protein functions by generating alternative C-termini. For example, intronic APA was previously shown to affect the molecular functions of *Homer1*, a gene that undergoes differential APA 3 hr post LTP induction. Our data is consistent with this finding (Fig. S5). The shorter *Homer1* CDS isoform that is inducible by neuronal activity acts in dominant negative fashion to inhibit the function of the full-length *Homer1* isoform^43^ by preventing dimerization. Here we revealed significant regulation of intronic APA in *Creb1*, which plays a key role in LTP^78^. Activation of intronic PAS of *Creb1* leads to a significantly truncated transcript that is likely to be rapidly degraded (see above). This mechanism may function to limit *Creb1*’s function in gene activation during LTP. Notably, activation of PAS in the first intron, as in the case of *Creb1*, is a widespread phenomenon during LTP. Further proteomic studies will provide insights into whether there is a surge of short peptides during LTP induction as a result of intronic APA activation, and, if so, whether they play functional roles in learning and memory.

## Methods

### Preparation of hippocampal acute slices

Hippocampal slices were prepared from isoflurane-anesthetized 2–3 month old male C57BL/6 mice (Charles River, Wilmington, MA, USA). Hippocampi were quickly isolated on ice, and 400-micron thick transverse slices were cut using a manual tissue chopper. The dentate gyrus was trimmed with a single incision, and the slices were placed in interface chambers at 30 °C to recover for 2 hr with continuous ACSF perfusion. From a single animal we collected ~10 hippocampal slices. Half of the mini-slices were used for cLTP induction and the remaining treated with a DMSO vehicle solution as time-matched controls. cLTP was induced by perfusing ACSF containing 50 uM forskolin for 5 min followed by 5 min of 50 uM forskolin, 30 mM KCl and 10 mM Ca^2+^ ACSF. Control slices were treated in parallel with ACSF containing 0.2% DMSO for 10 min. Slices were collected 1 hr or 3 hr post stimulation by freezing in dry ice. Use of mice in this study followed the recommendations of and protocol approved by the UCLA Institutional Animal Care and Use Committee. To validate successful LTP induction, we monitored expression of candidate transcripts by qPCR, and prepared sequencing libraries from samples that exhibited LTP-induced upregulation of *Arc* mRNA and increases in *Homer1* short isoform and unaltered concentrations of *Homer1* long isoform and *Hprt*. We obtained triplicates for all controls and cLTP samples for 3′READS+, RNA-seq and PAS-seq experiments.

### RNA extraction and RT-qPCR

To extract RNA, frozen slices were homogenized using a pestle in TRIzol for 5 min. 200 uL of chloroform per 1 mL of TRIzol were added to the homogenate and after centrifugation the top aqueous phase was collected. To precipitate and elute total RNA containing both mRNA and small RNAs we then used the Qiagen microRNeasy kit. We obtained ~2.5 ug of total RNA from 5 hippocampal slices. For RT-qPCRs we used 200 ng of total RNA and performed reverse transcription with random hexamers and SuperScript III in a total volume of 20 uL. The cDNA was then quantified by qPCR using SYBR green (primer sequences available upon request). The mRNA levels of *Hprt* were used as internal controls since its expression is activity-independent^79^.

### RNA-seq and Differential Expression Analysis

Three biological replicates of each condition (control and LTP-induced) at two different time-points (1 hr and 3 hr) after LTP were collected. For each replicate, hippocampal slices from the same animal were used to generate both LTP-induced samples and matched controls. The minimum RIN of all samples was 7.5, as determined by the 4200 Tapestation Instrument (Agilent). All RNA-seq libraries were prepared using the TruSeq Stranded Total RNA sample prep kit with Ribo-Zero according to manufacturer’s specified protocol (Illumina). Samples were multiplexed and sequenced across multiple HiSeq 2500 high-output lanes using 100 bp paired-end reads to achieve a minimum depth of ~75 M reads per sample. Transcriptome alignment was performed using STAR v. 2.4.1c^80^ with default settings, and GRCm38/mm10 (Data statistics, Table S2). Raw counts were quantified using R GenomicFeatures and GenomicAlignments and RSamtools packages^81^. Differential expression analysis was performed for each time-point separately in DESeq^82^. We excluded outlier samples, as determined by sample clustering (Fig. S1b). We applied an FDR cutoff of <0.1 and fold change >1.2 as the threshold for significance. GO analysis was carried out based on Fisher’s exact test.

### 3′READS+ and its analysis

The 3′ region extraction and deep sequencing (3′READS+) method was previously described in^44^. Briefly, 1 μg of input RNA was used for each sample, and poly(A) + RNA was selected using oligo d(T)25 magnetic beads (NEB), followed by on-bead fragmentation using RNase III (NEB). Poly(A) + RNA fragments were then selected using a chimeric oligo containing 15 regular dTs and five locked nucleic acid dTs conjugated on streptavidin beads, followed by RNase H (NEB) digestion. Eluted RNA fragments were ligated with 5′ and 3′ adapters, followed by RT and PCR (15x) to obtain cDNA libraries for sequencing on the Illumina platform. Processing of 3′READS+ data was carried out as previously described^12^. Briefly, reads were mapped to the mouse genome using bowtie 2^83^. Reads with ≥2 unaligned Ts at the 5′ end were used to identify PASs. PASs located within 24 nt from each other were clustered together (Table S1).

### APA analysis

Differential expression of APA isoforms are carried out with DEXSeq.^84^. Significant events were those with *p* < 0.05 and relative abundance difference >5%. Outlier samples were excluded as determined by sample clustering (Fig. S1a). Relative expression (RE) of two most abundant 3′UTR APA isoforms, e.g., proximal and distal PASs, was calculated by log2 (distal PAS/proximal PAS). Relative Expression Difference (RED) of two isoforms in two samples was based on difference in RE between the two isoforms in the two samples. For intronic APA analysis, RE was based on comparison of all intronic APA isoforms combined (intronic PAS set) with all 3′UTR PAS isoforms combined (3′UTR PAS set), and RED was also based on the two sets.

The weighted mean of 3′UTR size for each gene was based on 3′UTR sizes of all APA isoforms, weighted by the expression level of each isoform based on the number of PAS-containing reads.

### Analysis of introns

The intron location was based on the RefSeq database, considering all RefSeq-supported splicing isoforms. Introns were divided into five groups; first, second, last, second last and middle (contains all the introns between +2 and −2). Only genes with at least four introns were analyzed. Relative expression was calculated by intronic PAS read number divided by that of 3′UTR PASs of the same gene.

### Data access

The sequencing data from this study has been submitted to the NCBI Gene Expression Omnibus under SuperSeries accession number GSE84644 (RNA-seq, GSE84503; 3′READS+, GSE84643). RNA-seq and 3′READS data can also be accessed through a web based expression browser at https://coppolalab.ucla.edu/gclabapps/3readsbrowser/home.

Reviewer link: https://www.ncbi.nlm.nih.gov/geo/query/acc.cgi?token=efmjesqabfetlef&acc=GSE84644.

## Electronic supplementary material

Supplementary Information
