# Supplementary material for: Activity-Dependent Regulation of Alternative Cleavage and Polyadenylation During Hippocampal Long-Term Potentiation
Source: Sci Rep. 2017 Dec 12;7:17377. doi: 10.1038/s41598-017-17407-w (PMC5727029; doi:10.1038/s41598-017-17407-w)
Supplement: Supplementary file 1 — Supplementary Information [file 41598_2017_17407_MOESM1_ESM.pdf]

Supplementary Information

Activity-Dependent Regulation of Alternative Cleavage and Polyadenylation During Hippocampal Long-Term Potentiation

Mariana M. Fontes[1,2,#], Aysegul Guvenek[3,#], Riki Kawaguchi[4], Dinghai Zheng[3], Alden Huang[4], Victoria M. Ho[1,5], Patrick B. Chen[1,5], Xiaochuan Liu[3], Thomas J. O'Dell[6], Giovanni Coppola[4], *Bin Tian[3] & *Kelsey C. Martin[1,4]

[1]Department of Biological Chemistry, David Geffen School of Medicine, University of California, Los Angeles, Los Angeles, CA
[2]Graduate Program in Areas of Basic and Applied Biology, University of Porto, Porto, Portugal
[3]Department of Microbiology, Biochemistry and Molecular Genetics, Rutgers New Jersey Medical School, Newark, NJ
[4]Department of Psychiatry and Biobehavioral Sciences, Semel Institute for Neuroscience, David Geffen School of Medicine, University of California, Los Angeles, Los Angeles, CA
[5]Interdepartmental Graduate Program in Neuroscience, University of California, Los Angeles, Los Angeles, CA
[6]Department of Physiology, David Geffen School of Medicine, University of California, Los Angeles, Los Angeles, CA

[#]These authors contributed equally to this work.

***Corresponding Authors:**

Kelsey C. Martin E-MAIL: kcmartin@mednet.ucla.edu; TEL: (310) 825-5687
Bin Tian E-MAIL: btian@rutgers.edu; TEL: (973) 972-3615

Supplementary Tables

Table S1. Statistics of 3'READS data.

Treatment	Time	Replicate	Total Reads	Mapped Reads	PASS	PASS %	Genic Reads read #>2, relative abundance>=5%	Genic # of PAS	# of mRNA Genes	# of lncRNA Genes
Ctrl	1 hr	1	34,848,574	27,725,785	16,160,999	58.29	13,296,101	26,172	13,825	312
Ctrl	1 hr	2	37,893,332	28,830,344	15,754,344	54.65	12,954,097	25,885	13,731	313
Ctrl	1 hr	3	35,724,383	27,301,906	14,910,311	54.61	12,127,640	25,585	13,660	316
Ctrl	3 hr	1	34,336,951	27,021,000	14,533,107	53.78	11,978,476	25,760	13,705	300
Ctrl	3 hr	2	30,955,479	24,040,417	13,025,963	54.18	10,777,384	24,984	13,481	294
Ctrl	3 hr	3	24,257,204	20,137,227	13,329,374	66.19	10,713,874	26,684	13,863	330
LTP	1 hr	1	34,415,120	26,444,088	15,056,295	56.94	12,431,953	26,083	13,737	304
LTP	1 hr	2	35,384,159	27,504,487	15,041,935	54.69	12,353,179	25,844	13,727	314
LTP	1 hr	3	33,241470	23,641,517	12,027,623	50.88	9,792,437	24,908	13,445	306
LTP	3 hr	1	35,690,878	28,110,280	15,491,954	55.11	12,627,502	26,508	13,886	299
LTP	3 hr	2	32,684,202	25,946,636	14,309,010	55.15	11,822,957	25,807	13,656	304
LTP	3 hr	3	34,750,310	27,646,273	16,144,649	58.40	13,103,896	26,916	13,886	308

Poly(A) site-supporting (PASS) reads are those containing at least 2 non-genomic As at the 3'end.

Table S2. Statistics of RNA-seq data.

Treatment	Time	Replicate	Total Reads	Uniquely mapped Reads	Mapped reads %
Ctrl	1 hr	1	74,798,155	62,727,464	83.8
Ctrl	1 hr	2	102,624,047	84,770,513	82.6
Ctrl	1 hr	3	79,008,105	66,650,143	84.3
Ctrl	3 hr	1	88,073,394	74,098,617	84.1
Ctrl	3 hr	2	90,060,999	75,888,083	84.2
Ctrl	3 hr	3	102,083,620	83,818,708	82.1
LTP	1 hr	1	83,145,392	69,797,724	83.9
LTP	1 hr	2	77,250,609	64,010,343	82.8
LTP	1 hr	3	81,487,345	68,440,528	83.9
LTP	3 hr	1	89,125,216	75,920,310	85.1
LTP	3 hr	2	86,950,262	72,304,399	83.1
LTP	3 hr	3	94,701,352	77,544,850	81.8

Table S3. GO terms enriched for genes with shortened or lengthened 3'UTRs 3 hr post LTP.

GO Term	Category	-$\log_{10}$ *P*
**Enriched for 3'UTR shortened Genes, 3 hr post LTP**		
ossification	BP	5.4
negative regulation of photoreceptor cell differentiation	BP	4.7
regulation of gliogenesis	BP	4.4
positive regulation of smooth muscle cell proliferation	BP	4.4
hair follicle maturation	BP	4.3
nuclear lumen	CC	5.6
nuclear body	CC	3.3
**Enriched for 3'UTR lengthened Genes, 3 hr post LTP**		
negative regulation of lipid catabolic process	BP	2.4
cellular response to extracellular stimulus	BP	2.4
establishment of mitotic spindle localization	BP	2.3
response to activity	BP	2.3
energy homeostasis	BP	2.1
membrane-bounded organelle	CC	2.2
nuclear periphery	CC	2.0

BP, biological process; CC, cellular component. *P* is based on the Fisher's exact test.

Table S4. GO terms enriched for genes with activated or repressed intronic PAS 3 hr post LTP.

GO term	Category	-$\log_{10} P$
**Enriched for genes with activated intronic PAS, 3 hr post LTP**		
chaperone cofactor-dependent protein refolding	BP	3.6
neurotrophin signaling pathway	BP	3.2
lung saccule development	BP	3.0
cellular response to hepatocyte growth factor stimulus	BP	2.7
transcription initiation from RNA polymerase II promoter	BP	2.7
intracellular non-membrane-bounded organelle	CC	3.4
nucleoplasm	CC	2.8
**Enriched for genes with repressed intronic PAS, 3 hr post LTP**		
negative regulation of inflammatory response to antigenic stimulus	BP	3.6
ephrin receptor signaling pathway	BP	2.8
myeloid leukocyte differentiation	BP	2.7
cell-substrate junction assembly	BP	2.3
protein targeting	BP	2.1
signal recognition particle receptor complex	CC	2.5
laminin-3 complex	CC	2.3

BP, biological process; CC, cellular component. *P* is based on the Fisher's exact test.

Figure S1

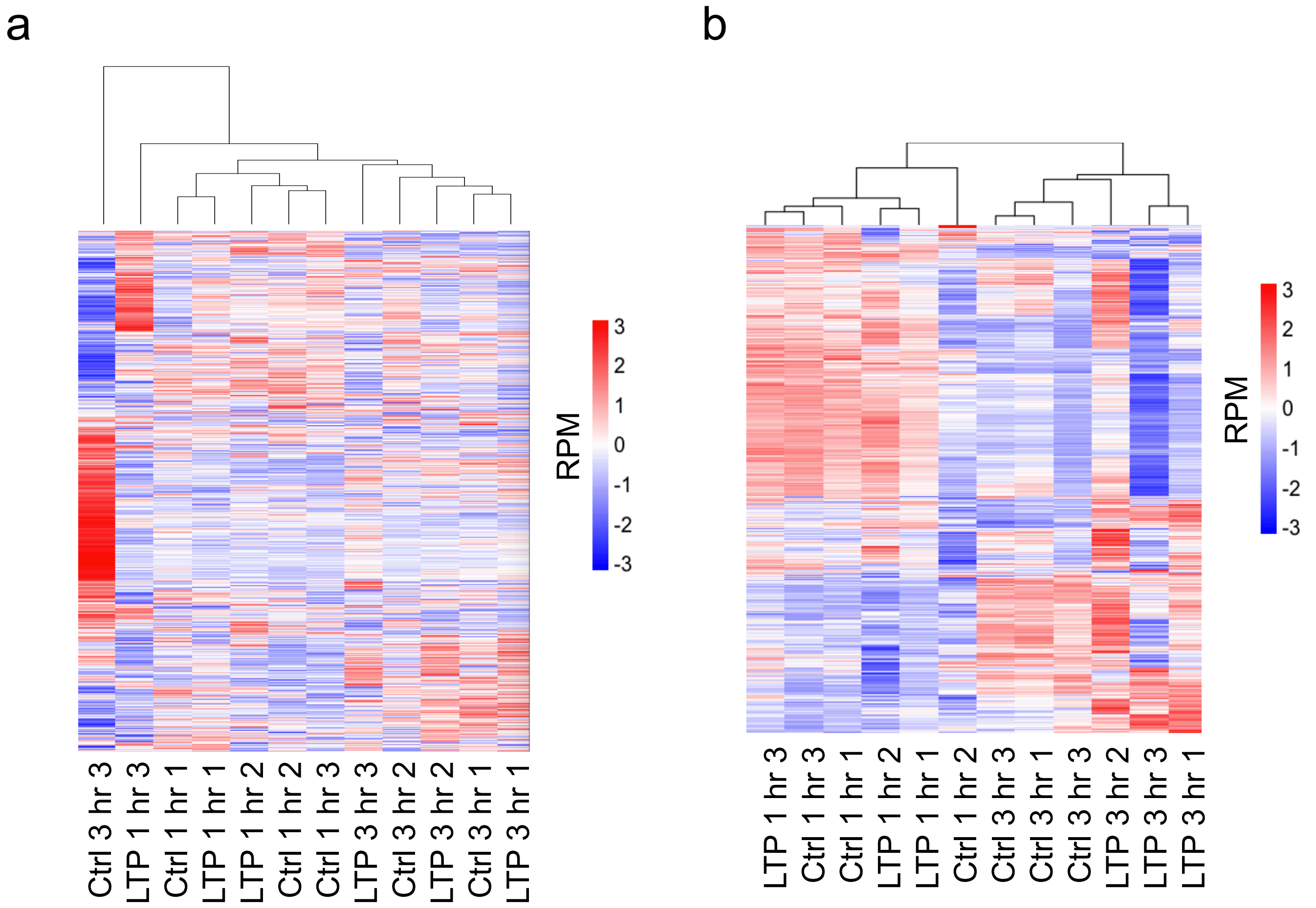

Figure S1. Clustering analysis of LTP and control samples. (a) RPM values (log2) of top 10,000 PASs were used. Control 3 hr sample #3 was identified as an outlier. (b) RPM values (log2) of top 2,000 genes were used. Control 1 hr sample #2 was identified as outliers.

Figure S2

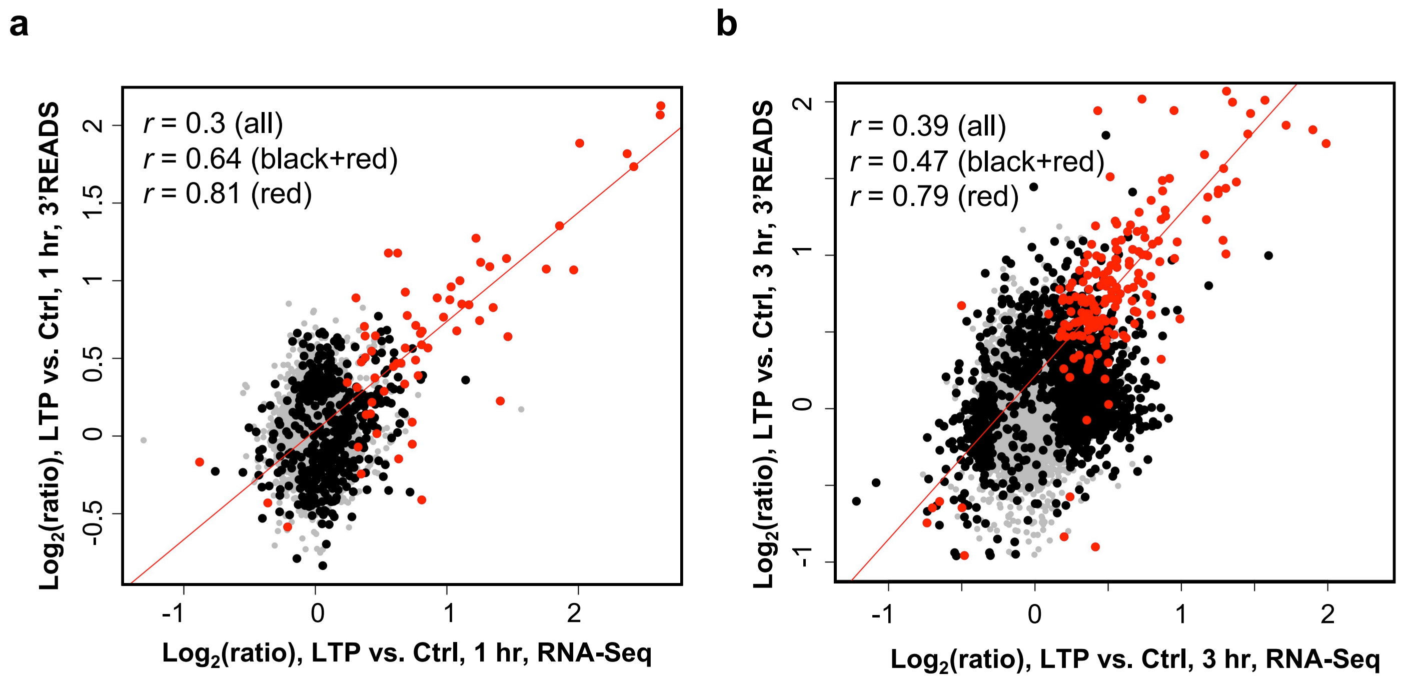

Figure S2. Correlation of gene expression between RNA-Seq and 3'READS in 1 hr (a) and 3 hr (b) samples. Number of genes analyzed is 9,746 or 9,998 for 1 hr or 3 hr sample, respectively. Each dot is a gene and red dots are genes that are commonly regulated in both datasets, black dots are genes that are significantly regulated in one of the datasets, and gray dots are genes not regulated in either dataset.

Figure S3

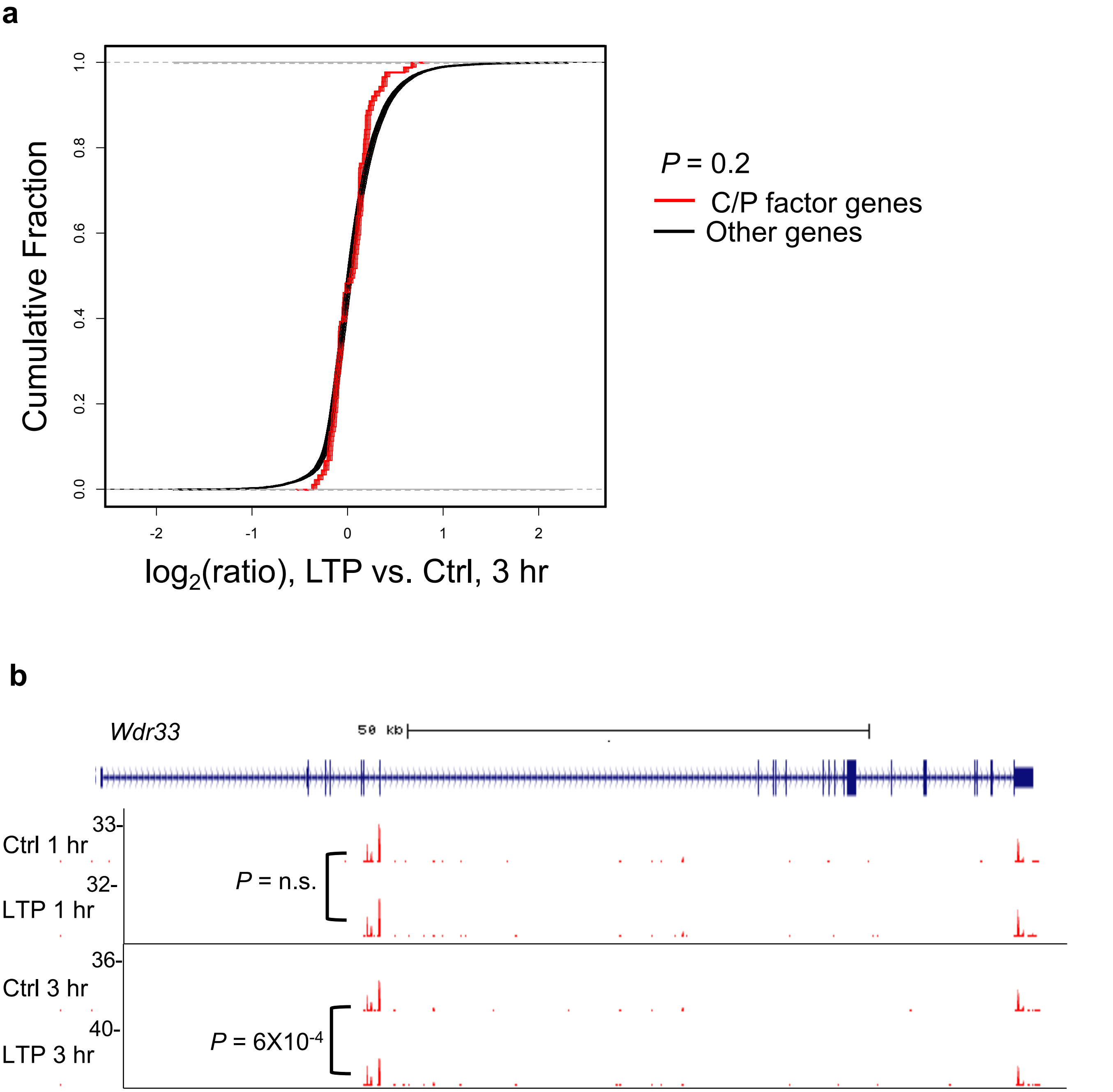

Figure S3. Regulation of C/P factors genes. (a) Log_2(ratio)(LTP vs. Ctrl) of C/P factor genes and other genes, red curve is 89 C/P factor genes in 3 hr post LTP sample and black curve is other genes in 3 hr post LTP. (b) Intronic APA regulation of *Wdr33*. *P*-values were based on DEXSeq, and read numbers are indicated. See Figure 2h for details about the figure format.

Figure S4

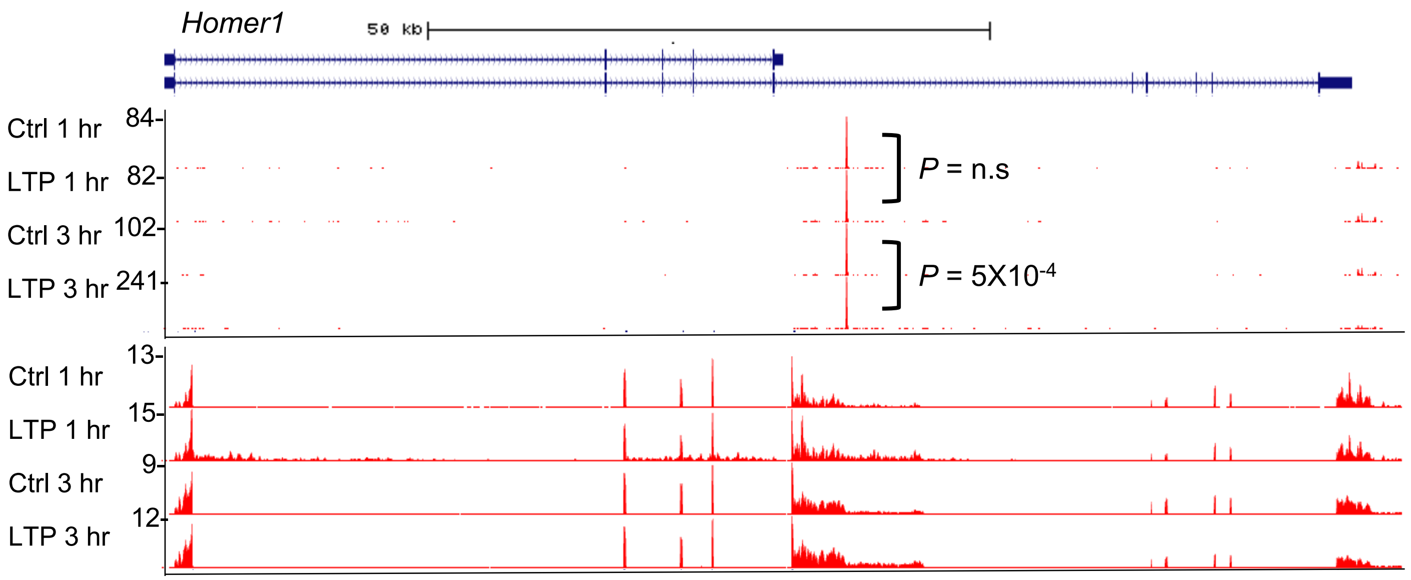

Figure S4. UCSC genome browser view of *Homer1*. *Homer1* is a gene with activated intronic PAS. Gene structure is on top and PAS peaks are shown in tracks. The top four tracks are 3'READS data and the bottom four are RNA-Seq data. *P-values* by DEXSeq are indicated for intronic PAS. Reads are based on combined samples.

Figure S5

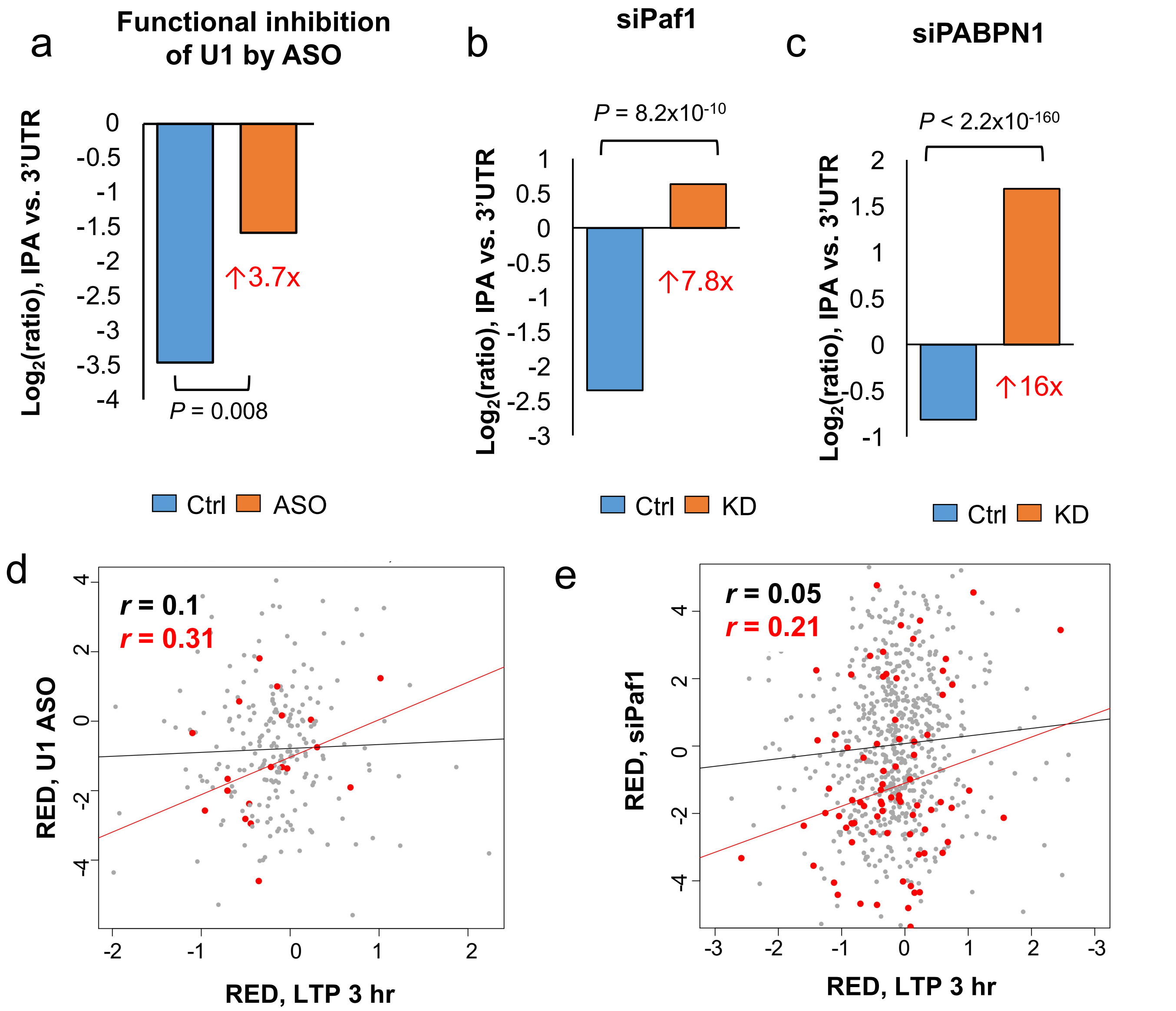

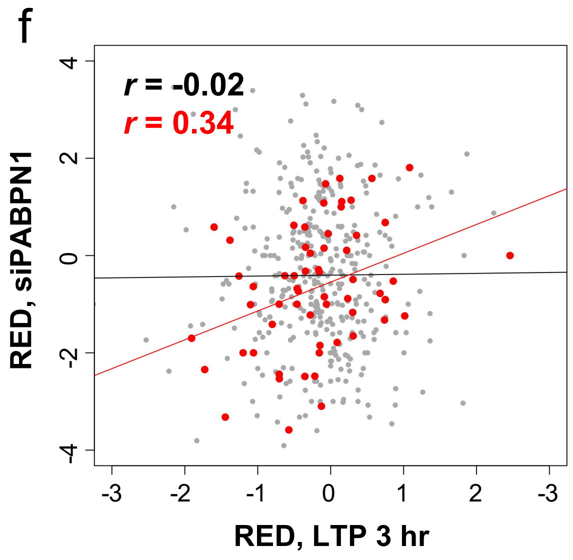

Figure S5. Regulation of *Creb1* and other intronic polyadenylation (IPA) events in C2C12 cells. (a-c) Data from Li et al.[15] and Yang et al.[56] for U1 inhibition by an antisense oligo (ASO) (a), siPABPN1 (b) and siPaf1 (c) were re-analyzed for *Creb1* IPA. Log2(ratio) of IPA expression vs. 3'UTR PAS expression are shown. P-values from the Fisher's exact test are indicated. Ratio of IPA increase is indicated. **(d-f)** Global comparison of RED values between LTP 3 hr and U1 inhibition (d), Paf1 knockdown (e) and PABPN1 knockdown (f) are shown in scatter plots. IPAs located in the first intron are highlighted in red. Pearson correlation values of all or first intron PAS are indicated on upper left corner of each plot in corresponding colors.
